# Bottleneck Analysis of Maternal, Newborn and Child Health Services in Underserved Areas of Kwale County, Kenya

**DOI:** 10.1177/11786329251374553

**Published:** 2025-09-24

**Authors:** Fatihiyya Wangara, Janne Estill, Hillary Kipruto, Caroline Perrin, Juma Ngudo, Khadija Nuru, Olivia Keiser

**Affiliations:** 1 Institute of Global Health, University of Geneva, Switzerland; 2 Department of Health Services, County Government of Kwale, Kenya; 3 Universal Health Coverage/Life Course Cluster, World Health Organization Regional Office for Africa, Brazzaville, Congo; 4 Department of Radiology and Medical Informatics, University of Geneva, Switzerland; 5 Health Systems Research Ethics Department, KEMRI-Wellcome Trust Research Programme, Kwale, Kenya

**Keywords:** bottleneck, antenatal, intrapartum, postnatal, access

## Abstract

**Background:**

Kenya experienced a 55% increase in maternal mortality between the years 2017 and 2020. While the global targets are yet to be realized, neonatal and infant mortality has improved over the years, but the rate of decline for neonatal mortality has been slow. The persistent high maternal mortality and slow improvements in neonatal and infant mortality warrant regular inquiries into health service provision, its quality and uptake.

**Objective:**

We assessed bottlenecks in accessing reproductive, maternal, newborn and child health (RMNCH) services in Kwale County, Kenya.

**Design:**

We used a cross sectional mixed methods approach.

**Methods:**

We adapted the Tanahashi model to evaluate RMNCH services using 5 key measures that reflect different stages along the service delivery continuum: availability of services; accessibility; initial contact with the health system; continued utilization; and quality coverage. Secondary quantitative data was collected from the Kenya Demographic and Health Survey 2022, Kenya Health Facility Census Report 2023 and other peer reviewed publications. Primary qualitative data was collected from 20 focus group discussions with 176 members including lay community members, community health promoters (CHPs) and traditional birth attendants. Primary data was collected over a 1 month period, between October and November 2022.

**Results:**

The main bottleneck identified from the supply side was the limited number and negative attitude of the healthcare workers. Access to core health workers was at 13/10 000 people, lower than the national average and World Health Organization (WHO) recommendation. From the supply side, low health literacy, gender norms and financial constraints were the major factors fueling the poor health seeking behavior.

**Conclusion:**

Kwale County needs to prioritize investments in human resources for health, advocacy, communication and social mobilization.

## Introduction

Despite most maternal deaths being preventable, it is estimated that globally, a maternal death occurs every 2 minutes.^1^ The Sustainable Development Goals include reducing the global maternal mortality ratio to <70/100 000 live births, the neonatal mortality ratio to <12/1000 live births and the under-5 mortality ratio to <25/1000 live births.^2^ Progress has been uneven between and within countries, with disparities favoring the wealthy and urban populations.^3^ Africa had a maternal mortality ratio (MMR) of 531 deaths/100 000 live births and accounted for 69% of global maternal deaths in 2020. This MMR is 8 times higher than the global target. Countries in Africa show varying trends in MMR, with an increase in MMR in 17 countries between 2017 and 2020.^1^

Eight African countries were categorized by the WHO as having a very high MMR. These include Central African Republic (835), Guinea-Bissau (725), Liberia (652), Lesotho (566), Guinea (553), Democratic Republic of the Congo (547), Kenya (530) and Benin (523). The MMR in Kenya of 530 deaths/100 000 represents a 55% increase between the year 2017 and 2020.^1^ While the global targets are yet to be realized, neonatal and infant mortality has improved over the years, but the rate of decline for neonatal mortality has been slow.^4^ According to the Kenya Demographic and Health Survey 2022, the neonatal mortality rate (NMR) and infant mortality rate (IMR) were 19 and 23 deaths/1000 live births, respectively.^5^ Neonatal period was defined as the first 28 days of life while infants were children aged below 1 year. The persistent high MMR and slow improvements in NMR and IMR warrant regular inquiries into health service provision, its quality and uptake. For instance, over 80% of maternal deaths have been attributed to poor quality of care.^6^ A 2019 study involving 81 low and middle income countries concluded that closing the quality gap while holding levels of access and utilization constant would lead to reduction in mortality by 21% to 32%. Interventions offered around the time of childbirth were most critical, and accounted for 64% of the overall impact.^7^

The Tanahashi framework,^8^ originally proposed in 1978, is a conceptual model used to assess and improve health service coverage, particularly in terms of accessibility (initial contact with the health system and continued utilization), quality and effectiveness. It provides a systematic approach to identifying barriers and gaps in the delivery of healthcare services, to ensure that interventions reach and benefit the target population.^9^ This model, including its modified versions has been widely used and applied by several organizations like UNICEF, World Bank and WHO to promote universal health coverage.^8,10,11^ While national data on RMNCH services exist in Kenya, there is a lack of in-depth subnational analyses that address specific contextual barriers in underserved regions such as Kwale County. Using the “Tanahashi model” we aim to assess supply and demand side bottlenecks for select RMNCH services including antenatal, intrapartum and postnatal care as well as childhood immunization.

## Methods

### Study Setting

The study was conducted in Kwale County, on the Kenyan Coast. Kwale is 1 of the 47 counties in Kenya and 1 of the 6 counties that form the coast region. In the year 2022, Kwale had an estimated population of 969 359, with 23.7% of them being women of reproductive age (15-49 years). The estimated numbers of pregnant women and live births were 230 000 and 36 400, respectively.^12^ According to the Kenya Demographic and Health Survey (KDHS) 2022, Kwale had a NMR of 19/1000 live births and an IMR of 23/1000 live births. The use of modern contraception was at 34.6% while unmet need for family planning among married women was 24.4%.^5^

### Study Design

We used a cross sectional mixed methods approach. First, we conducted a quantitative assessment of health service determinants as per the domains of the Tanahashi model. Quantitative data was in the form of percentages and were extracted from desk review. We then conducted focus group discussions with lay community members, community health promoters (CHPs) and traditional birth attendants that aimed to assess more details on the observed coverage of key RMNCH interventions.

### Data Sources

Both primary (qualitative) and secondary (quantitative) data sources were collected. Quantitative data was used to estimate service coverage and utilization. This was then triangulated with qualitative data to understand reasons behind the estimated coverage and utilization of RMNCH services. The quantitative variables described in Table 1 were collected through a review of the Kenya Demographic and Health Survey 2022, Kenya Health Facility Census Report 2023 and other peer reviewed publications.

**Table 1.** Description of Variables and Their Sources.

Variable	Description
*Source:* MoH^15^	
Pharmaceuticals	The mean availability of 28 tracer drugs in Kenya. The Kenyan Ministry of Health uses the term tracer drugs to refer to a select few essential drugs. A list of these essential drugs is provided in Supplement 1. The national estimate was used as a proxy due to gaps in inventory management at health facilities within the county, characterized by incomplete documentation and hence unreliable data
Human resource	Core health workforce (medical officers, dental officers, pharmacists, nurses and clinical officers) access per 10 000 people. The population numbers were based on the 2019 Kenya Population Census data. The WHO recommended ratio is 23/10 000 population
*Source:* Moturi et al^35^	
Geographic access	The proportion of population accessing care within 1 h of the nearest facility (includes public health facilities and those run by faith based and non-governmental organizations) at county level. Several modes of transport were considered within a single journey between the household and the nearest health facility. The assumption was that one walks to the nearest health facility without access to motorable roads or through motorized transport if a motorable road was adjacent to a residence and connected to a health facility.
*Source:* KNBS^5^	
First ANC	Percentage of women aged 15 to 49 y who had a live birth and/or a stillbirth in the 2 y preceding the survey, who received at least 1 ANC session from a skilled provider (doctors, nurses, midwives or clinical officers) for the most recent live birth or stillbirth
Fourth ANC	Percentage of women aged 15 to 49 y who had a live birth and/or a stillbirth in the 2 y preceding the survey, who received 4 or more ANC sessions from a skilled provider (doctors, nurses, midwives or clinical officers) for the most recent live birth or stillbirth
Skilled delivery	Among all live births and stillbirths in the 2 y preceding the survey, percentage delivered by a skilled provider
PNC within 48 h	Percentage of women aged 15 to 49 y with a live birth or stillbirth in the 2 y preceding the survey, who received a postnatal check during the first 2 d after giving birth
BCG	Percentage of children aged 12 to 23 mo who had received the vaccine at any time before the survey, as indicated either on the child’s vaccination card or reported by the mother
DPT-HepB-Hib1	
DPT-HepB-Hib3	
FIC (basic antigens)	Percentage of children aged 12 to 23 mo who received specific vaccines at any time before the survey (according to a vaccination card or the mother’s report). Children were considered fully vaccinated against all basic antigens if they had received the BCG vaccine, 3 doses each of polio vaccine (excluding OPV given at birth) and DPT containing vaccine, and a single dose of measles-containing vaccine
FIC (national schedule)	Percentage of children aged 12 to 23 mo who received specific vaccines at any time before the survey (according to a vaccination card or the mother’s report). A child was considered to be fully vaccinated according to the national schedule if the child had received all basic antigens as well as a birth dose of OPV, a dose of IPV, 3 doses of the pneumococcal vaccine, and 2 doses of the rotavirus vaccine.

Abbreviations: ANC, antenatal care; BCG, *Bacillus* Calmette-Guérin; DPT, diphtheria-pertussis-tetanus; FIC, fully immunized child; HepB, hepatitis B; Hib, *Haemophilus influenzae* type b; IPV, inactivated polio vaccine; MR, measles-rubella 1; OPV, oral polio vaccine; OPV 0, polio vaccination given at birth; PNC, postnatal care.

### Procedure

Qualitative data was collected over a 1 month period between October and November 2022. Focus group discussions (FGDs) were structured to cover diverse perspectives by including 4 categories of participants; adult male community members, adult female community members, community health promoters (CHPs) and traditional birth attendants (TBAs). This was a convenience sample and participants needed to have lived in Kwale county for at least 3 years. Via phone calls, we approached local administrators to assist with selection of adult community members. Health facility staff selected CHPs and TBAs. The local administrators and the health facility staff approached the participants face-to-face. The FGD guides were pilot tested among each participant category. A total of 38 participants were included in the pilot and their feedback incorporated to improve the tools. The 38 pilot FGD participants were a distinct group and were not part of the larger FGD sample. Topics that were discussed included service availability, accessibility, utilization and respective recommendations. FGDs were conducted in various locations to capture diverse regional insights and covering both urban and rural regions. The FGDs were conducted within health facilities’ compounds. No other member(s) was present besides the participants and the researchers. FGDs were facilitated by the first (female) and fifth (male) authors using semi-structured discussion guides included in Supplement 2. Both interviewers had Master’s degree level of training in a health related field and worked within the department of health services in Kwale County. Before the start of each FGD, the interviewers introduced themselves and gave more insight into the study. Each participant category had 5 FGDs of 7 to 12 members that achieved concept saturation as no new themes emerged during the final FGDs. Each discussion took between 60 and 90 minutes. Discussions were held in Kiswahili. No repeat focus groups were carried out.

### Qualitative Data Synthesis

**Figure 1.**
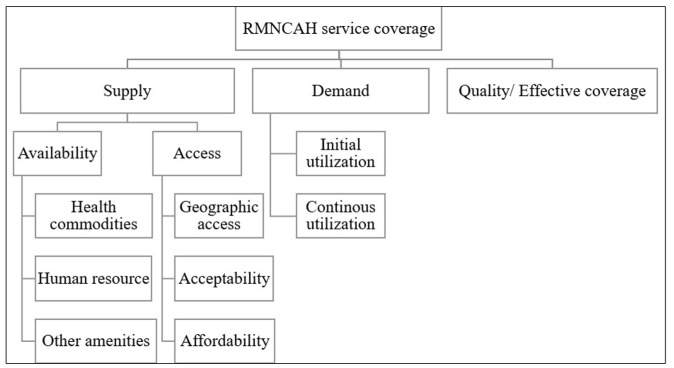
Coding tree for thematic analysis.

Qualitative data was analyzed through thematic content analysis in Microsoft Excel. The transcription was verbatim; afterwards, coding was done by 2 independent data coders via a blended approach including both deductive and inductive coding.^13^ Predefined codes were according to the Tanahashi model. Through inductive coding, additional codes were included as illustrated in Figure 1.

### Quantitative Data Analysis

Using Microsoft Excel, quantitative data on the different indicators were summarized in frequencies and percentage coverage, and the cascade was displayed in bar graphs. In line with the Tanahashi framework, we first looked at the lowest determinant(s) in the supply side then at the bigger drops in the cascade within the demand side and quality.^14^

### Ethics

The study was approved by an ethics review board (reference number ERC/PhD/010/2021). Informed verbal consent was sought from all FGD participants and their identity anonymized throughout the study. Verbal consent was preferred as it was more acceptable to the participants and the study had minimal risk. The consent was simultaneously captured on audio recording and in the researchers’ notes. Upon completion of the study, the findings were shared during a stakeholder’s meeting with the view of informing S/RMNCAH programming.

## Results

**Figure 2.**
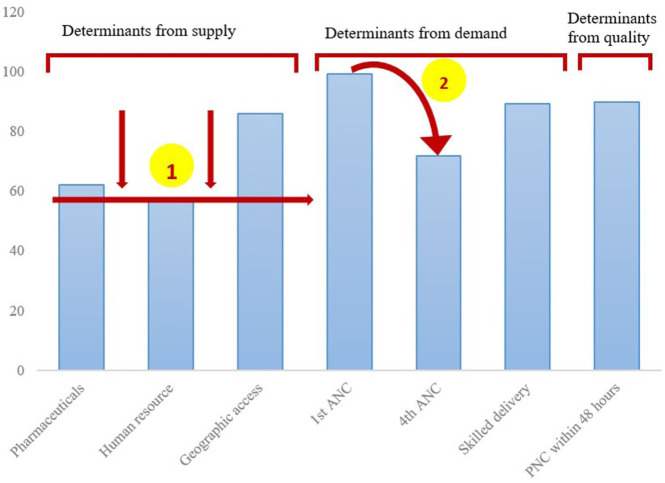
Access to maternal and newborn continuum of care in Kwale County, Kenya. ANC, antenatal care; PNC, postnatal care. The arrows and numbers illustrate the areas to prioritize, that is, 1, the lowest determinant(s) in the supply side; 2, biggest drop in the demand side cascade.

**Figure 3.**
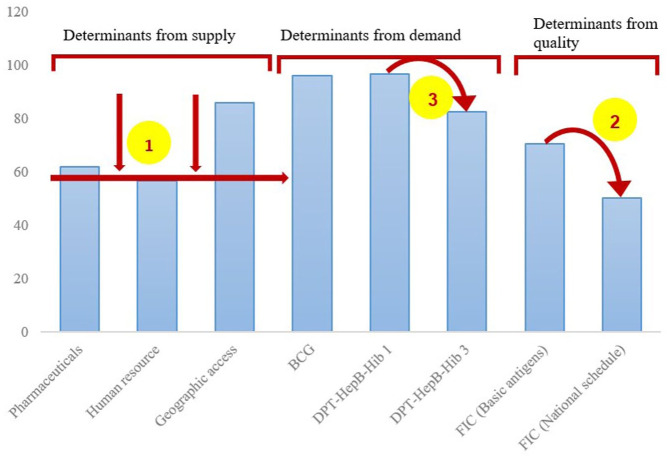
Access to essential childhood immunizations in Kwale County, Kenya. BCG, *Bacillus* Calmette-Guérin; DPT, diphtheria-pertussis-tetanus; FIC, fully immunized child; HepB, hepatitis B; Hib, *Haemophilus influenzae* type b. The arrows and numbers illustrate the areas to prioritze, that is, 1, the lowest determinant(s) in the supply side; 2, biggest drop in the demand side cascade; 3, second biggest drop in the demand side cascade.

In line with the Tanahashi framework, we first looked at the lowest determinant(s) in the supply side then at the bigger drops in the cascade within the demand side and quality.^14^ The main bottleneck to RMNCH service access was limitations in human resources. Bigger drops in the demand side cascade were between the first and fourth ANC; DPT-HepB-Hib1 and DPT-HepB-Hib3; and uptake of all vaccines as per the national schedule. The results are summarized in Figures 2 and 3.

Twenty FGDs were conducted with a total of 176 participants, ensuring geographical representation across the study area. Of the 176 participants, 62 (35%) were male and 114 (65%) were female. The participants’ age ranged from 15 to 58 years, with 84% of them aged between 15 and 49 years. The literacy level was low, with only 57% of the participants having received some level of formal education. Table 2 summarizes numbers of FGD participants by participant category and geographical location (Sub County). Qualitative and quantitative data were triangulated to provide a more in-depth understanding of the results.

**Table 2.** Number of FGD Participants by Participant Category and Geographic Location.

Participant category	Sub county					Total
	Kinango	Lungalunga	Matuga	Msambweni	Samburu	
Adult females	9	9	10	8	11	**47**
Adult males	9	11	8	7	8	**43**
Community health volunteers	8	8	8	8	8	**40**
Traditional birth attendants	10	9	12	8	7	**46**
Total	**36**	**37**	**38**	**31**	**34**	**176**

## Supply Side Determinants

### Pharmaceuticals

According to the Kenya health facility census 2023, the mean availability of tracer drugs (see Supplement 1) was 62%.^15^ The participants of the health facility census described drug stock outs as frequent, and detrimental to the community mobilization efforts by the CHPs. Kwale county offers free health services in all level II (dispensaries) and level III (health centers) health facilities, resulting in an influx of clients from the neighboring county (Mombasa city) and subsequent high commodity consumption at the health facilities near the border. Besides the tracer drugs, the community desired food and nutritional support specifically for persons living with HIV (PLHIV).

### Human Resource

Core health workforce access was 13/10 000 people, lower than the national average and WHO recommendation of 20 and 23 core health workers/10 000 population, respectively. Thus, the health worker coverage was 56.5% of the WHO target.^15^ The participants’ primary concern was the inadequate numbers of health care workers leading to long waiting times and sometimes lack of services. Misconduct among health workers was also highlighted, with some being rude, negligent and prioritizing familiar clients, disregarding the principles of triage:

A woman can arrive at a dispensary at 10 am but leave at 4 pm due to limited HCWs. (Participant 9, adult female)

There’s a specific day for ANC, every Thursday. But in case a delivery comes in, everyone else has to wait or sometimes miss services. One time the nurse was awake the whole night monitoring labour, then two deliveries came in the morning. Staff was too sleepy and she referred the women to another facility at 2 pm. Sometimes 1 staff has to man ANC, PNC and maternity. Busy clinic days forces staff to be too quick with clients. Even the community pity the staff. (Participant 21, CHP)

Amidst the shortage of technical staff, there has been a constant need for task shifting, with CHPs taking up additional roles within the health facilities. The participants emphasized the need to adequately train CHPs before shifting technical tasks like drug dispensing. There was however high attrition of CHPs due to lack of stable remuneration and/or incentives. While the CHPs met monthly to interrogate data and review performance, the health facility in-charge was often unavailable, hence a missing link between the community and the health facility. Traditional birth attendants (TBAs) still influence the access to obstetric care for women in Kwale. Despite clear communication on the range of services to be offered by TBAs (client referral, birth preparation, escorting unaccompanied women), they still monitor labor and conduct deliveries when necessary, especially for babies born before arrival to a health facility. The TBAs cited a lack of recognition by CHPs and HCWs as a major cause of demotivation, considering their invaluable inputs towards the care of mothers and their newborns:

We do important work but the relationship between TBAs and CHPs is that of a goat and a leopard. I once witnessed dogs pulling a newborn’s cord. I assisted, clamped the cord and saved the baby. I even named the baby. I then cleaned the baby, baby was weighed and a clinic card issued. Because of shortage of health workers, sometimes we monitor a woman in labor as the health worker sleeps. We call the HCW when necessary. (Participant 34, TBA)

TBAs also reported that they occasionally assisted HCWs to monitor labor. During the FGDs, the TBAs requested for several items to help improve their efficiency, that is, a form of identification (badges), gloves, torches and closed shoes for fear of snake bites during night referrals. Other incentives desired by the TBAs included regular trainings, acknowledgement by HCWs, regular meetings with health facility staff and special consideration for relief food distribution.

Other identified bottlenecks that did not fit well into the major themes of the Tanahashi model included water shortage, inadequate bed capacity, lack of observation rooms in some health facilities and power blackouts.

### Accessibility

Accessibility to MNH services was analyzed across 3 dimensions: geographic access, acceptability and affordability. In Kwale County, ~86.4% and 94.0% of the population lived within 1 and 2 hours to a health facility, respectively. This implies that 6% of the population were marginalized as far as MNH services were concerned.^16^ Several modes of transport were considered within a single journey between the household and the nearest health facility. The assumption was that one walks to the nearest health facility without access to motorable roads or through motorized transport if a motorable road was adjacent to a residence and connected to a health facility. Excluding privately owned health facilities, 85.9% and 93.6% of the population lived within 1 and 2 hours to a health facility, respectively. About 70% and 90% of the population lived 1 and 2 hours from a private health facility, respectively.

The community felt that the health facilities were closely located. They however emphasized the importance of looking at geographic access in relation to the levels of care. For instance, the majority of the health facilities were dispensaries that operated only on weekdays from 8 am to 4 pm and lacked laboratory and/or ultrasound services. While outreaches and home visits by CHPs bridged the gaps whenever necessary, there was a bias in selection of the CHPs by village elders and subsequent discrimination of households (HHs) by the CHPs. Due to HCW shortage, only CHPs may attend outreaches, negating the purpose of the integrated outreach. The referral system was weak, characterized by delays attributed to human resource inefficiencies, and worsened by a poor road network. There were isolated cases of flooding, insecurity and wildlife attacks especially during night referrals:

Referral by ambulance works from one health facility to another but not from a household. Additionally, the ambulances serve host facilities well but not other catchment areas. If CHP is well known to the ambulance driver or HCW, then referral is smooth. Sometimes there is no fuel for the ambulance. Sometimes we use motorbikes. The CHPs tell us that motorbikes injure the baby whereas TBAs say they help the baby descend. (Participant 64, Adult female)

In Kwale County, not all health facilities have an ambulance. Host facilities are health facilities that have an ambulance and are tasked with the responsibility of managing ambulance operations, to also neighboring catchment areas with no ambulances.

Family planning services were resisted because of side effects and religious beliefs, while cervical cancer screening was less accepted when offered by male staff. The acceptability of RMNCAH services by the community is summarized in Table 3.

**Table 3.** Acceptability of RMNCAH Services.

Service	Reason(s) for unacceptability
Family planning	• Side effects, for example, dizziness, missed periods or bleeding thrice a month, abdominal pain, loss of libido believed to be due to frequent family planning • Fear that family planning would cause a miscarriage • Religious beliefs coupled with disapproval by the male partner
Cervical cancer screening	• When the screening is offered by a male staff
Antenatal care	• Deliberate delays in initiating first ANC in order to reduce the number of ANC visits • The belief that “pregnancy is no illness” • Women may need men’s approval to attend clinics. Very few men are supportive during pregnancy; supportive men are termed as cowards • Men fear HIV testing • Stigma for HIV positive women in waiting rooms. The separate queues for HIV positive clients fuels stigma and discourages service uptake • Clients often seek TBAs’ green light before accepting specific tests, for example, ultrasounds
Skilled delivery	• Some women were shy to deliver within the local dispensary • While most women did not mind to be attended to by male nurses, the male partners were uncomfortable with the exposure of their wives’ nakedness • Culturally, the placenta needed a special burial ceremony, where it should be buried facing upwards. This was believed to enhance child bearing in the future as opposed to the standard disposal of placentas at the health facilities. Disposal in the placenta pit was believed to cause secondary infertility. The participants suggested that HCWs should consider asking individual women whether they would like to carry their placentas with them • Denying the TBAs access into the delivery room discouraged skilled delivery
Postnatal care	• For babies’ hygiene, mothers trusted TBAs more than HCWs, leading to inappropriate practices, for example, the belief that water caused asthma hence babies were cleaned with coconut oil instead of water
CWC	• The belief that some vaccines, for example, polio vaccine caused paralysis and cancer • Upon completing the immunization schedule, most mothers did not see the value of attending CWC besides knowing the baby’s weight • Mothers whose children were living with HIV and had persistent low weight faced stigma at CWC
Medication	• Fear of drug side effects • More faith in traditional medicine, fueled by illiteracy • Unpalatable drugs, for example, antimalarial • The belief that sweet tasting “modern medicine” is not as effective as bitter medicine • The belief that frequent drug use causes congenital anomalies • Seeing no value; “. . . In the older days, there were no drugs and still, people lived” • Religious sects that were against seeking medical services, for example, the “miracle church” used sand to treat wounds and relied on God for divine intervention
HH visits by CHPs	• CHPs were sometimes denied entry into households due to lack of identifying badges • A less receptive community that values food distribution more than health products and messages
All services	• Inadequate services for persons living with disability, for example, the deaf/dumb/mentally handicapped cases needed an escort or else, they were faced with communication challenges at health facilities

Abbreviation: CWC, child welfare clinic.

### Affordability

Kwale County offered relatively affordable health services. All dispensaries and health centers offered free health services to clients of all ages. The 5 hospitals in the county charged a subsidized fee for consultation (about US$1) and separate charges for subsequent services. Children aged below 5 years received free services across all levels of care, including the hospitals. Additionally, there was a national insurance cover dubbed “*Linda mam*” (Swahili phrase meaning protect the mother) offered to all women, covering antenatal care, skilled delivery, postnatal care, conditions and complications during pregnancy and infant care.^17^ Critics of this insurance however claim that:

The president only cares about pregnant women, children and the elderly. (Participant 84, adult male)

The community however highlighted related costs that negatively affect access to health services. These include transport costs that were higher for persons living with disability; referral costs for laboratory services and unavailability of HCWs in dispensaries after 4 pm and over the weekend. This forces them to seek services from private health facilities which translates to higher costs.

## Demand Side Determinants

### Initial Utilization

The community was well aware of the importance of seeking timely RMNCAH services. While almost all pregnant women (99%) attended at least 1 ANC visit^5^ they deliberately delayed their first visit in order to avoid multiple visits to the health facility prior to delivery. Other disadvantaged groups included pregnant teenagers and single mothers who shied away from attending clinics due to stigma and the emphasis on male partner involvement. Both men and women feared taking an HIV test, which was a requirement at first ANC or at first contact. Some men did not see the value of ANC due to lack of incentives, for example, relief food. Additionally, the FGD participants stated that polygamy is widely practiced in Kwale and it was not uncommon for men to neglect pregnant wives and marry other wives. This neglect often led to poor uptake of health services. Motivators of ANC uptake included referral by the CHP and any form of abdominal discomfort.

### Continuous Utilization

The big gap between initial ANC (99%) and fourth ANC (72%) coverage was due to the deliberate delays in ANC uptake, causing many pregnant women to deliver before they could attend at least 4 ANC visits. Coverages of skilled delivery and PNC were 89% and 90%, respectively. Often, women were unsure of the gestation, contributing to home or unskilled deliveries. However, women were aware of postpartum hemorrhage as a potential complication of child birth, motivating them to seek skilled delivery services. Reluctance to seek healthcare and low health literacy were cited as the main reasons for dropouts during the postnatal period, especially among women who had other older healthy children to whom these health services were never offered.

Approximately 96% of children had received the BCG vaccine, whereas the coverages for DPT-HepB-Hib1 and DPT-HepB-Hib3 were 97% and 83%, respectively. Erratic supply of vaccines forced HCWs to schedule vaccinations, sometimes with frequent change of appointment dates. The participants did not see the value of vitamin A administration and (child welfare clinic) CWC attendance, claiming that the latter was only useful in measuring the babies’ weight, something they could easily do at home. Other barriers to continuous utilization of postnatal and child health services include refusal by male partners and having several children aged below 5 years hence higher transport/logistics costs. In some health facilities, HCWs would decline to offer PNC, CWC and outpatient services to mothers who delivered at home. This led to either attending private health facilities or missing services altogether:

The HCWs are unfriendly. At outpatient, doctors refuse to attend to children who have not attended CWC. There are no health services for children without clinic card. No services for children who are delivered at home. Staff tell women that if you deliver in private facility, then go to private facility for subsequent clinic. Lack of clinic card can even lead to denial at school enrolment. So, there is a chemist that charges Ksh 20 to weigh baby and fills MCH booklet. (Participant 101, Adult female)

### Quality/Effective Coverage of Maternal, Newborn and Child Health Services

Reliable metrics for quality were not available from secondary data for the stated study period. As such, proxy indicators were used. For maternal and newborn services, effective coverage was defined as receiving PNC within 48 hours. About 90% of the target population received PNC services within 48 hours of childbirth. For immunization, we defined effective coverage as the percentage of children aged 12 to 23 months who received all vaccines as required by Kenya’s Expanded Programme on Immunization (EPI), which includes the BCG vaccine, birth dose of OPV, a dose of IPV, 3 doses of the pneumococcal vaccine, 2 doses of the rotavirus vaccine, 3 doses each of polio vaccine (excluding OPV given at birth) and, DPT containing vaccine, and a single dose of measles-containing vaccine. The greatest area of concern in immunization was the many missed opportunities for OPV at birth, vaccination against pneumonia and rotavirus, with only 50% of the target population being fully vaccinated as per the national schedule.

## Discussion

Our data shows that human resources (number of healthcare staff) should be the main focus area if supply of health services is to improve within Kwale County. While Kenya has made significant strides in human resources for health (HRH) across most counties, challenges remain in ensuring that the training and production of HRH meets the needs of the health system and maintains quality of care. Nationally, only 12 out of 47 counties had the required number of core health workers (nurses, clinical officers, doctors) per population as per the WHO recommendations.^18^ This staff shortage has necessitated task shifting and/or task sharing with CHPs, sometimes without clear guidelines. Studies in Democratic Republic of Congo (DRC) have shown that the presence of community health workers (CHWs) improved health service delivery, despite staffing and retention challenges.^19^ In Tanzania, the CHWs proved effective in promoting male participation in maternal and child health issues.^20^

Despite the National Reproductive Health Policy (2007) that required all TBAs to stop providing child birth services and instead, accompany pregnant mothers to health facilities,^21^ TBAs in Kwale County still conduct deliveries for babies born before arrival in a health facility. In many LMICs, TBAs continue to offer obstetric care to women but express their desire for recognition by the formal health system.^22-24^ A study in South Africa showed that collaboration between midwives and TBAs expanded the reach and improved outcomes of community health care.^22^ Proposed approaches to strengthening the working relations include knowledge/practice sharing, trainings, bilateral referral systems, inclusion of TBAs in outreach programs and approving TBAs as birth companions. Other programs like the SHE (Skilled Health Entrepreneur) program in Bangladesh^23^ and the “*Agbebiye*” program in Nigeria^24^ engaged TBAs through economic empowerment, skill acquisition workshops and formal linkage to the health system. The “*Agbebiye*” program drastically reduced TBA-related maternal mortality.

Having proactive staffing policies is key to address HRH challenges. Some recommended approaches include recruiting staff from among underserved communities and rural areas; implementing provider payment reforms and emphasizing the positive impact of the work done.^19^ These have been shown to improve staff motivation, attitude, compassion and retention. Many studies have shown that caring and sympathetic health providers who were familiar with patient’s cultural practices, encouraged demand and utilization of ANC.^20^

Stock outs for essential pharmaceuticals and medical supplies affects health service delivery in many African countries. Further, disparities between urban and rural areas persist. This calls for more equitable resource allocation^20^ and innovative funding models. A study in Tanzania showed that “pay for performance” models have the potential to increase the availability of essential health products and technologies (HPT).^20^ Other proven interventions to improve HPT availability include having parallel supply chains, buffer stocks and a decentralized commodity storage.^19^

Studies have shown an association between the place of residence and ANC attendance within the first trimester of pregnancy.^20,25^ While geographic accessibility was relatively good in Kwale, major challenges persisted when other dimensions of access were considered; availability of service, affordability and acceptability. Out of the 170 government owned health facilities serving a population of about 900 000 people in Kwale county, there was only 1 level 5 (County referral hospital), 4 level 4 (sub county hospitals) and 10 level 3 (health centers) facilities, while the rest were level 2 health facilities (dispensaries). As such, only 5 health facilities offered comprehensive emergency obstetric care. There were no level 1 health facilities. Rather, level 1 health services refers to services offered at household level by CHPs. Delays in receiving appropriate care due to inadequate skilled health workforce, inadequate medical equipment and poor referral mechanisms has been shown to contribute to the high maternal mortality in the African Region. Lack of transport to tertiary facilities has also been linked to the causes of maternal mortality in Sub-Saharan Africa.^1^ Strengthening referral networks and facilitating geographic access to health care through inclusive universal health coverage (UHC) policies, innovative transportation methods, mobile clinics and health posts has been shown to improve access to healthcare in Africa.^19^ While the use of technology like telemedicine for remote consultations is becoming more popular, studies show that people living in developing countries still prefer face-to-face meeting with healthcare providers. Additionally, there is also a perceived risk in embracing telemedicine.^20^

Acceptability of health services is largely influenced by cultural believes and social/gender norms. For the delivery of effective RMNCH services, it is essential that all services along the continuum of care are culturally appropriate.^26^ In many African countries, women are expected to prioritize their family’s health over their own.^27^ Empowering women to be able to move away from restrictive social norms would improve access to healthcare as demonstrated by a study in Myanmar,^28^ where women in rural areas were disproportionately affected. Additionally, gender norms have been shown to hinder men’s participation in pregnancy and childbirth. Being the decision makers at home, men negatively impact the timing of ANC in Malawi.^29^ Given that culture and social norms vary greatly among communities, including cultural skills and sensitivity training in health education curriculum is vital.^30^

While no fees were charged at primary healthcare facilities in Kwale county, transport and referral costs was a barrier to accessing care. Reducing referral costs could be achieved by ensuring health facilities are well equipped to offer services at their respective levels. As such, the health department should be run in a way to ensure sustainability. Research shows that, to achieve UHC, system-wide reforms are inevitable. These include defining and costing the UHC package; increasing government spending for health services, particularly from domestic revenue sources; flat fees or social health protection schemes; introducing results-based planning and subsequent results-based budgeting.^11,30^

Initial service contact was relatively good but there was high attrition across the RMNCH continuum of care, specifically at ANC and childhood immunization. Studies have shown that the higher the educational level of a woman, the more likely they are to fully attend ANC.^31,32^ Some strategies reported to effectively improve immunization uptake relate to adjusted hours for and timing of immunization services, tailoring delivery to meet client needs, peer support for health workers and reminder call systems.^33^

Socioeconomic inequalities is the main barrier to effective coverage of RMNCH services in low and middle income countries (LMICs). These inequalities are worse in rural areas and in countries with low Human Development Index (HDI). A study using data from 39 LMICs revealed that socioeconomic inequalities are more evident in outcomes that are difficult to achieve, such as user adherence.^25^ There is thus a need to actively monitor quality of care indicators in addition to the coverage indicators. A study in Nepal established that women who received good quality RMNCH care came from advantaged ethnic groups, had access to bank accounts, had a low birth order (<4) and did not have a preference for female healthcare providers. Additionally, women who received better quality ANC services had higher odds of receiving optimal quality institutional delivery.^34^

This study has several strengths. We utilized population level quantitative data from the demographic Health Survey and health facility census. As opposed to health service uptake data at health facility level, population level data gives a better representation of the community’s needs and health seeking behavior. Triangulation with qualitative data provides an in-depth understanding of the bottlenecks. This study was however not without limitations. Due to resource constraints, only qualitative data was primarily collected and secondary quantitative data was used to estimate health service coverage,^5^ geographic accessibility^35^ and health facility readiness.^15^ As such, sample size/ power analysis was not performed. The secondary data were collected at different time points during the years 2022 and 2023. The assumption was that service coverage, location of health facilities and facility service readiness did not vary greatly over the data collection period. Additionally, although the indicators assessed are useful in assessing health services, they do not reflect the content or quality of the care provided or the extent to which key interventions are implemented as intended.^36^ There are also caveats to data interpretation given the definition of variables as summarized in Table 1. For example, a child was captured as immunized based on the record on the vaccination card or as reported by the mother. This may introduce recall bias and/or deliberate misrepresentation of the child’s immunization status by the mother.

To improve access to RMNCH services, Kwale County should prioritize HRH recruitment, training and explore other incentives to motivate staff. There is also need to address attrition among CHPs and exploring areas of effective collaboration with TBAs as per the National Reproductive Health Policy. Reviewing the approach for HPT resource allocation and considering parallel supply chains may be of value in addressing the stock outs experienced. Lessons learnt from other LMICs can be of benefit in improving access to health services. Some key strategies to consider include in-service training for cultural skills and sensitivity; sustained health education; strengthening the referral network while reducing referral costs; investing in effective and efficient client follow up mechanisms, specifically during the antenatal period and 6 to 14 weeks postnatal. Long term solutions would revolve around increasing government spending on health; registration of beneficiaries on social health protection schemes; results-based planning and multi-sector approaches to alleviate poverty and improve livelihoods.

## Supplemental Material

sj-docx-1-his-10.1177_11786329251374553 – Supplemental material for Bottleneck Analysis of Maternal, Newborn and Child Health Services in Underserved Areas of Kwale County, Kenya Supplemental material, sj-docx-1-his-10.1177_11786329251374553 for Bottleneck Analysis of Maternal, Newborn and Child Health Services in Underserved Areas of Kwale County, Kenya by Fatihiyya Wangara, Janne Estill, Hillary Kipruto, Caroline Perrin, Juma Ngudo, Khadija Nuru and Olivia Keiser in Health Services Insights

sj-docx-2-his-10.1177_11786329251374553 – Supplemental material for Bottleneck Analysis of Maternal, Newborn and Child Health Services in Underserved Areas of Kwale County, Kenya Supplemental material, sj-docx-2-his-10.1177_11786329251374553 for Bottleneck Analysis of Maternal, Newborn and Child Health Services in Underserved Areas of Kwale County, Kenya by Fatihiyya Wangara, Janne Estill, Hillary Kipruto, Caroline Perrin, Juma Ngudo, Khadija Nuru and Olivia Keiser in Health Services Insights

sj-docx-3-his-10.1177_11786329251374553 – Supplemental material for Bottleneck Analysis of Maternal, Newborn and Child Health Services in Underserved Areas of Kwale County, Kenya Supplemental material, sj-docx-3-his-10.1177_11786329251374553 for Bottleneck Analysis of Maternal, Newborn and Child Health Services in Underserved Areas of Kwale County, Kenya by Fatihiyya Wangara, Janne Estill, Hillary Kipruto, Caroline Perrin, Juma Ngudo, Khadija Nuru and Olivia Keiser in Health Services Insights
