# Supplementary material for: Bottleneck Analysis of Maternal, Newborn and Child Health Services in Underserved Areas of Kwale County, Kenya
Source: Health Serv Insights. 2025 Sep 24;18:11786329251374553. doi: 10.1177/11786329251374553 (PMC12461047; doi:10.1177/11786329251374553)
Supplement: sj-docx-1-his-10.1177_11786329251374553 – Supplemental material for Bottleneck Analysis of Maternal, Newborn and Child Health Services in Underserved Areas of Kwale County, Kenya [file sj-docx-1-his-10.1177_11786329251374553.docx]

**Supplement 1: A list of “tracer drugs”. Source- Kenya Health Facility Census Report 2023.**

Olanzapine

Fluoxetine

Midazolam Injection 5mg/ml, 3ml

Premixed Insulin

Loratadine Tablets, 10mg

Soluble insulin

Carbamazepine 200mg

Amlodipine 5mg

Benzyl penicillin Injection 5 MU

Benzyl penicillin Injection 1 MU

Magnesium Sulphate Injection,…

Chlorhexidine gel, 7.1%…

Metformin 500mg

Oxytocin Injection 10 I.U.

Gentamicin Injection, 40mg/2ml

Amoxicillin Dispersible Tablets, 250mg

Paracetamol Syrup/Suspension,…

Sodium hypochlorite solution 4-6%

Nystatin oral suspension 100IU/ml

Tetracycline Eye Ointment,…

Amoxicillin Capsules, 500mg

Adrenaline Injection 1mg/ml

Metronidazole Tablet, 400mg

Sodium chloride, 0.9% (isotonic),…

ORS Co-Pack…

Hydrocortisone Injection 100mg

Albendazole Tab. 400mg

Paracetamol Tablets, 500mg
