# Supplementary material for: Bottleneck Analysis of Maternal, Newborn and Child Health Services in Underserved Areas of Kwale County, Kenya
Source: Health Serv Insights. 2025 Sep 24;18:11786329251374553. doi: 10.1177/11786329251374553 (PMC12461047; doi:10.1177/11786329251374553)
Supplement: sj-docx-2-his-10.1177_11786329251374553 – Supplemental material for Bottleneck Analysis of Maternal, Newborn and Child Health Services in Underserved Areas of Kwale County, Kenya [file sj-docx-2-his-10.1177_11786329251374553.docx]

**SUPPLEMENT 2: SEMI-STRUCTURED FGD GUIDES**

**FOCUSED GROUP DISCUSSION GUIDE: ADULT BENEFICIARIES**

1. **Note to the interviewer:** For each FGD participant, note the gender, age and residence.
2. Introduction and purpose of the discussion
3. Obtain informed consent (verbal)
4. How would you describe access and uptake of health services in your community?

- *Probe: distance to health facilities and reasons for utilisation (or lack of) health facilities*
- *Antenatal clinic attendance for pregnant women. At what stage of the gestation do they report to a health facility and how frequently? Why do you think this is so?*
- *Where do women deliver their babies and why?*

1. Do pregnant women access and utilise health services for the antenatal purposes (and not necessarily because they feel unwell)?

- *Probe if the service providers are skilled or unskilled*

1. In Kwale, many women drop out of the continuum of care and do not complete post natal care attendance. Why do you think this is so? How can this be improved?
2. Do you think there are certain pregnant women who are unable to access antenatal, maternity and postnatal care service? Why do you think they are disadvantaged and what can be done and by whom to improve this situation?
3. Do men play any additional roles to support their wives when pregnant?

- *Probe further for reasons of support or lack of*

1. Do you think the health care services offered to women and children is adequate? Why or why not?

- *Probe: What are some of the woman’s worries related to the health of their babies?*

1. How can you describe the health referral system i.e. from community level to higher levels of care? How can this be improved?

- *Probe on the relationship between beneficiaries and community health volunteers; also probe on availability of ambulance services*

1. What would you consider the most appropriate methods for health education in this area and why?
2. What things can you, as an individual do to improve health status in your community?
3. What things do you feel should be done by the local and national authorities to improve health status in your community?

**Our discussion has ended, if you have any other questions/comments related to this topic you make ask.**

**Thank you for your time.**

**FOCUSED GROUP DISCUSSION GUIDE: COMMUNITY HEALTH PROMOTERS (CHPs)**

1. **Note to the interviewer:** For each FGD participant, note the gender, age and residence.
2. Introduction and purpose of the discussion
3. Obtain informed consent (verbal)
4. Explain to us your role in maternal and child health care programs

- *Explore the roles at* ***community*** *and* ***facility*** *level****;*** *including recording & reporting, mentorship*

1. Describe how adequately or inadequately the MCH program has been able to serve the vulnerable groups

- *Explore services for the special groups; pregnant & lactating women, the hard reach populations, persons living with disability, displaced persons)*

1. Mention to the CHPs some of the MCH indicators that seem to indicate good signs of progress and find out from them what could have led to this

- *First ANC attendance; immunization coverage*

1. Similarly mention the indicators that are indicating poor performance and discuss the possible reasons

- *≥ 4 ANC attendance; skilled delivery generally and skilled delivery among HIV positive women; postnatal care attendance*

1. Do you feel that the needs of pregnant women and their newborns are adequately addressed by the department of health services? If not, what health needs should be of focus?
2. What are some of the barriers to access and utilization of health services by the community?

- *Probe on geographical barriers, social & cultural barriers, economic barriers)*

1. How can you describe the health referral system i.e. from community level to higher levels of care? How can this be improved?
2. How would you describe the monitoring and evaluation of community health services?

- *Probe on availability of community referral forms, service delivery logbooks; report submission mechanisms and feedback; analysis of data and data use; role of CHAs; how planning for community activities is normally done)*

1. What things can you, as a CHP or as a community health unit (CHU) do to improve the health status in your community?
2. What things do you feel should be done by the local and national authorities to improve health status in your community?

**Our discussion has ended, if you have any other questions/comments related to this topic you make ask.**

**Thank you for your time.**

**FOCUSED GROUP DISCUSSION GUIDE: TRADITIONAL BIRTH ATTENDANTS (TBAs)**

1. **Note to the interviewer:** For each FGD participant, note the gender, age and residence.
2. Introduction and purpose of the discussion
3. Obtain informed consent (verbal)
4. What type of services do you offer to the community members?

- *Probe roles at antenatal, delivery and postnatal*

1. Do you think it is important for pregnant women to seek services at a health facility? If so, why do you think they still seek the services of TBAs? If you do not consider it important, why so?
2. Are there cases where you have had to refer clients to a health facility? What was the reason for referral? What was your experience?

- *Probe if and how they contacted the health facility staff before referring the client; mode of transport of the client to the health facility; any feedback from the health facility)*

1. How would you describe your relationship with the health facilities? Do you feel this relationship should be improved and if so, how?

- *Probe if TBAs have received any form of training from MoH; how does the referral system work; incentives for client referral*

1. In Kwale, many women drop out of the continuum of care and do not complete post natal care attendance. Why do you think this is so? How can this be improved?
2. Do you think there are certain pregnant women who are unable to access antenatal, maternity and postnatal care service? Why do you think they are disadvantaged and what can be done and by whom to improve this situation?
3. Do men play any additional roles to support their wives when pregnant?

- *Probe further for reasons of support or lack of*

1. What would you consider the most appropriate methods for health education in this area and why?
2. What things can you, as an individual do to improve the health status in your community?
3. What things do you feel should be done by the local and national authorities to improve health status in your community?

**Our discussion has ended, if you have any other questions/comments related to this topic you make ask.**

**Thank you for your time.**
